# Determinants of treatment and outcomes of diverticular abscesses

**DOI:** 10.1186/s13017-019-0250-5

**Published:** 2019-07-08

**Authors:** Juha Mali, Panu Mentula, Ari Leppäniemi, Ville Sallinen

**Affiliations:** 1 Department of Abdominal Surgery, University of Helsinki and HUS Helsinki University Hospital, Haartmaninkatu 4, 00029 HUS, Helsinki, Finland; 2 Department of Transplantation and Liver Surgery, University of Helsinki and HUS Helsinki University Hospital, Helsinki, Finland

**Keywords:** Diverticulitis, Abscess, Drainage, Antibiotics

## Abstract

**Background:**

Diverticular abscess diameter of 3–6 cm is generally accepted as a cutoff determining whether percutaneous drainage is recommended in addition to antibiotics, but this is not based on high-quality evidence. The aim of this study was to analyze the treatment choices and outcomes of patients with diverticular abscesses.

**Methods:**

This was a retrospective cohort study conducted in an academic teaching hospital functioning as a secondary and tertiary referral center. Altogether, 241 patients with computer tomography-verified acute left-sided colonic diverticulitis with intra-abdominal abscess were collected from a database containing all patients treated for colonic diverticulitis in our institution during 2006–2013. The main measured outcomes were need of emergency surgery and 30-day mortality, and these were compared between antibiotics only and percutaneous drainage groups. Treatment choices, including surgery, were also analyzed for all patients.

**Results:**

Abscesses under 40 mm were mostly treated with antibiotics alone with a high success rate (93 out of 107, 87%). In abscesses over 40 mm, the use of emergency surgery increased and the use of antibiotics alone decreased with increasing abscess size, but the proportion of successful drainage remained at 13–18% regardless of the abscess size. There were no differences in failure rate, 30-day mortality, the need of emergency surgery, permanent stoma, recurrence, or length of stay in patients treated with percutaneous drainage vs. antibiotics alone, even when groups were adjusted for potential confounders.

**Conclusions:**

Percutaneous drainage as a treatment for large abscess does not seem to be superior to the treatment with only antibiotics.

## Background

Diverticular disease of the colon is a common ailment, especially among the elderly, present in approximately 65% of the population over 65 years of age [1]. However, only 5% of the patients with diverticular disease develop an acute diverticulitis during their lifetimes [2]. Most of the episodes of acute diverticulitis are uncomplicated, but 15–20% of those diagnosed with computed tomography (CT) imaging are complicated by an intra-abdominal abscess [3, 4]. Due to their rarity, the treatment of diverticular abscesses is not based on high-quality evidence.

Abscess size of 3–6 cm is generally accepted as a reasonable cutoff determining the choice of treatment [5–10]. World Society of Emergency Surgery guidelines recommend antibiotics alone for abscesses with a diameter less than 4–5 cm [11]. Some studies have suggested that the smallest abscesses might be safely treated with only oral antibiotic in an outpatient setting or possibly even without antibiotics [12, 13]. Percutaneous drainage of the abscess combined with intravenous antibiotics is recommended for larger abscesses, but the evidence to support this is of low quality [11]. No randomized controlled trials comparing the treatment of diverticular abscesses using drainage with antibiotics to antibiotics alone exist. However, data from retrospective series suggest an approximately 20% failure rate for both drainage with antibiotics and antibiotics alone [14]. Emergency surgery is usually reserved for unstable patients or patients not responding to conservative treatment as it is associated with higher mortality (12% vs. 1.1% if treated non-operatively) [11, 14]. However, this excess mortality might be more due to the selection bias and unmodifiable factors (sepsis or comorbidities) than the surgery itself. The aim of this study was to analyze the treatment choices and their outcomes for diverticular abscesses of different sizes.

## Methods

This was a retrospective cohort study conducted in HUS Helsinki University Hospital, which is an academic teaching hospital functioning as a secondary and tertiary referral center for a population of 1.7 million. ICD-10 code K57 query for years 2006–2013 produced 2780 patients treated for diverticular disease. Screening of electronic patient records identified 1514 patients with either intraoperatively or CT-verified acute colonic diverticulitis. Of these, 264 had CT-verified diverticular abscess. Data was extracted manually from the electronic patient records, and parameters regarding age, comorbidities, laboratory tests, imaging studies, treatment, and recurrent diverticulitis were collected. Recurrences within 30 days of discharge were considered as the same episode of diverticulitis.

CT imaging criteria of diverticular abscess were bowel wall thickening, fat stranding, inflamed diverticulum, and presence of an intra-abdominal abscess in relation to diverticulitis. On-call radiologist (resident or attending) analyzed the CT images, and later, an attending radiologist re-evaluated the images. Resident or attending surgeon at the emergency department either admitted patients to the hospital or, if clinical condition permitted, discharged them with per oral antibiotics. The most commonly used antibiotics were metronidazole combined with either cefuroxime or cefalexin for intravenous or per oral treatment, respectively. If deemed necessary, the surgeon requested percutaneous drainage. On-call radiologist evaluated the amenability of an abscess to drainage and placed drain with CT or ultrasound guidance. There were no strict departmental guidelines regarding drainage. If the patient required emergency surgery, on-call surgeon (always consultant level of expertise) made the decision to operate based on the clinical condition, laboratory parameters, and radiological findings.

Patients diagnosed with colonic cancer mimicking diverticulitis, either during surgery or after routine follow-up colonoscopy, were excluded from the study. Limitation of treatment to conservative means based on the patient’s wishes or comorbidities and living outside the referral area of HUS Helsinki University Hospital were also exclusion criteria. Failure of treatment was defined as death or need of operative treatment during the initial admission or within 30 days of discharge. In the antibiotics group, the need for drainage during the initial admission or within 30 days of discharge was also considered a failure.

SPSS Statistics 24 (IBM, Armonk, NY) was used for statistical analysis. Mann-Whitney *U* test, Kruskal-Wallis test, *χ*^2^ test, chi-square linear-by-linear association, and Fisher exact test were used where applicable. A multivariate logistic regression model was created to determine the independent risk factors for failure of treatment. This study was approved by an institutional review board.

## Results

**Fig. 1:**
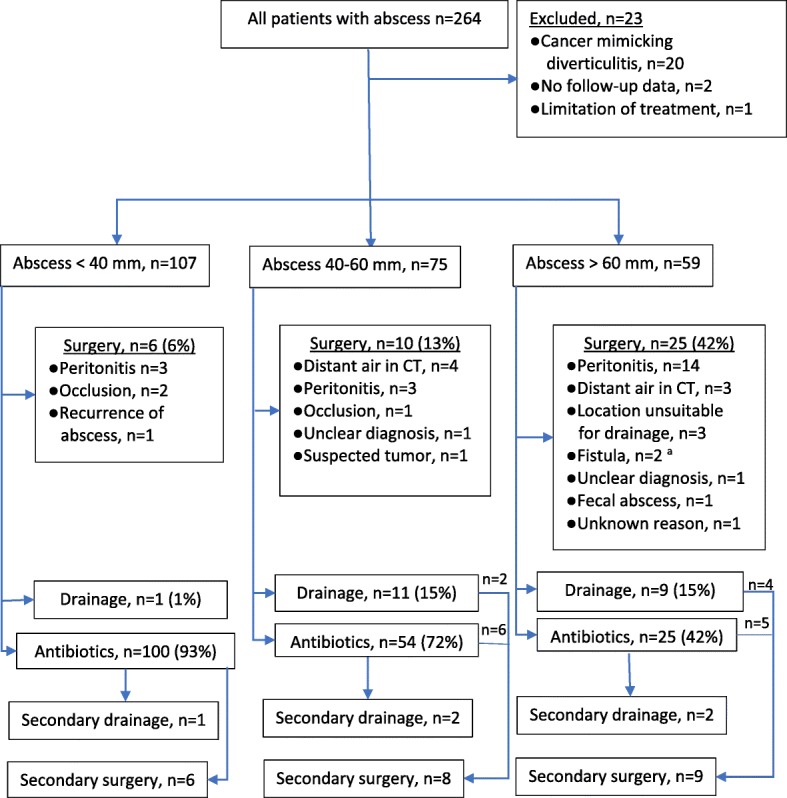
Flowchart of the primary and secondary treatment choice during index admission categorized by abscess size. Reasons for first-line operative treatment are also listed. ^a^One colovesical and one enterocutaneous fistula

Overall, 264 patients with CT-verified acute left-sided colonic diverticulitis with intra-abdominal abscess were evaluated for the study, and 241 were included in the analyses after exclusions (Fig. 1). Ten (4%) patients had a recurrent diverticular abscess. Median time from earlier diverticular abscess to recurrence for these patients was 150 days (interquartile range (IQR) 72–335 days). Altogether, 17 (7%) patients were treated as outpatients. Patients were divided into groups for every 20 mm increase in the largest diameter of the abscess. C-reactive protein (CRP) level on admission and Charlson Comorbidity Index were higher, and the use of glucocorticoid medication was more frequent among patients with larger abscesses (Table 1).

**Table 1:** Basic characteristics and outcomes for patients grouped by the diameter of the largest abscess

	< 20 mm (*n* = 25)	20–39 mm (*n* = 82)	40–59 mm (*n* = 71)	60–79 mm (*n* = 35)	≥ 80 mm (*n* = 28)	*p* value
Basic characteristics						
Sex female	17 (68%)	48 (59%)	43 (61%)	18 (51%)	19 (68%)	0.64^a^
Age, years, median (IQR)	63 (57–68)	59 (48–69)	61 (50–73)	65 (59–73)	68 (58–77)	0.13^b^
WBC, × 10^9^/l, median (IQR)	11.4 (8.4–14.7)	11.7 (9.5–14.5)	12.0 (9.0–15.0)	13.8 (7.2–16.0)	13.2 (9.1–17.2)	0.73^b^
CRP, mg/l, median (IQR)	139 (109–200)	117 (80–159)	131 (86–210)	216 (106–273)	190 (128–288)	0.008^b^
Earlier diverticulitis	5 (20%)	26 (32%)	20 (28%)	7 (20%)	5 (18%)	0.46^a^
Multiple abscesses	0	13 (16%)	14 (20%)	6 (17%)	3 (11%)	0.18^a^
Charlson Comorbidity Index, median (IQR)	2 (1–3)	2 (1–3)	2 (1–4)	3 (1–5)	3 (1–5)	0.02^b^
Glucocorticoid medication	1 (4%)	5 (6%)	10 (14%)	7 (20%)	5 (18%)	0.01^c^
Pelvic abscess	2 (8%)	9 (11%)	28 (39%)	21 (60%)	18 (64%)	< 0.001^c^
Outcomes						
Antibiotics only as first-line treatment	25 (100%)	75 (91%)	53 (75%)	16 (46%)	10 (36%)	< 0.001^c^
Antibiotics only successful	24 (96%)	68 (91%), *n* = 75	43 (81%), *n* = 53	12 (75%), *n* = 16	3 (30%), *n* = 10	< 0.001^c^
Drainage attempted^d^	0	5 (6%)	17 (24%)	9 (26%)	9 (32%)	< 0.001^c^
Received drain^d^	0	0	9 (13%)	2 (6%)	7 (25%)	< 0.001^c^
Aspiration only^d^	0	2 (2%)	2 (3%)	3 (9%)	1 (4%)	0.18^c^
Successful drainage^d^	0	2 (100%), *n* = 2	9 (82%), *n* = 11	4 (80%), *n* = 5	5 (63%), *n* = 8	0.002^c^
Operative treatment^e^	1 (4%)	12 (15%)	17 (24%)	17 (49%)	20 (71%)	< 0.001^c^
Hartmann	0	8	8	10	12	
Primary anastomosis	1	3	6	6	5	
Drainage operatively	0	1	2	1	2	
Colectomy	0	0	1	0	1	
Operative treatment as first line	0	6 (7%)	9 (13%)	14 (40%)	12 (43%)	< 0.001^c^
Successful first line operative treatment^f^	0	6 (100%), *n* = 6	9 (100%), *n* = 9	8 (57%), *n* = 14	10 (83%), *n* = 12	0.15^c^
30-day mortality	0	1 (1%)	3 (4%)	7 (20%)	1 (4%)	0.01^c^
Length of hospital stay, days, median (IQR)	2 (1–3)	3 (1–6)	4 (3–9)	6 (4–13)	15 (6–25)	< 0.001^b^

*WBC* white blood cell count, *IQR* interquartile range, *CRP* C-reactive protein

^a^*χ*^2^ test

^b^Kruskal-Wallis *H* test

^c^Linear-by-liner *χ*^2^ test

^d^Either as first-line treatment or after failed treatment with antibiotic

^e^Either as first-line treatment or after failure of conservative treatment

^f^No re-operation or death within 30 days

The diameter of the largest abscess ranged from 11 to 169 mm, and therefore, treatment strategies differed considerably. Operative treatment was a primary strategy for 41 (16%) patients based on clinical or radiological findings, most commonly due to the clinical peritonitis or radiological distant intraperitoneal air (Fig. 1). Majority of the operatively treated patients (93%) underwent Hartmann’s procedure or sigmoidectomy with primary anastomosis (Table 1). Antibiotics alone, either oral or intravenous, was the predominant treatment (100 of 107, 93%) for patients with an abscess smaller than 40 mm (Fig. 1).

**Fig. 2:**
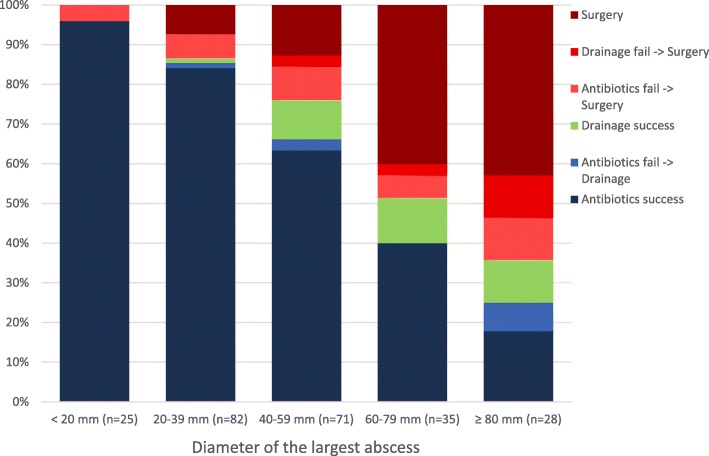
Percentages of the first-line treatment choice and results categorized by the diameter of the largest abscess

The proportion of patients that required operative treatment, either primarily or after failed conservative treatment, increased as the abscess size increased (Fig. 2). However, the percentage of successfully drained abscesses remained the same (13–18%) for all groups with abscess over 40 mm (Fig. 2). Also, the portion of patients that were successfully treated with antibiotics alone decreased as the abscess size increased (Table 1).

The results of the first-line treatment with either antibiotics alone or combined with percutaneous drainage were compared for abscesses of 40 mm or larger. Only 1% of smaller abscesses under 40 mm were treated by drainage. The drainage group had slightly larger abscess diameter (median 60 mm vs. 51 mm), and this was the only difference between the groups in basic characteristics (Table 2). No differences were noted in overall failure rate, 30-day mortality, need of emergency surgery within 30 days, recurrences, later elective sigmoidectomy rate, or in need of permanent stomas (Table 2). Median follow-up time was 71 months (IQR 46–100 months).

**Table 2:** Basic characteristics and treatment results for patients with abscess diameter ≥ 40 mm and for matched patients

Abscess ≥ 40 mm	Antibiotics (*n* = 79)	Drainage (*n* = 20)	*p* value	Matched antibiotics (*n* = 18)	Matched drainage (n = 18)	*p* value
Basic characteristics						
Sex female	50 (63%)	8 (40%)	0.06^a^	11 (61%)	7 (39%)	0.18^a^
Age, years, median (IQR)	61 (52–73)	60 (48–69)	0.52^b^	67 (55–78)	60 (50–69)	0.15^b^
WBC, × 10^9^/l, median (IQR)	13.6 (10.1–15.6)	11.1 (8.5–13.9)	0.10^b^	12.0 (9.2–15.3)	10.5 (8.4–13.1)	0.26^b^
CRP, mg/l, median (IQR)	140 (97–231)	126 (44–277)	0.79^b^	162 (50–235)	110 (39–270)	0.45^b^
Any earlier diverticulitis	20 (25%)	8 (40%)	0.19^a^	6 (33%)	8 (44%)	0.49^a^
Multiple abscesses	15 (19%)	2 (10%)	0.51^c^	1 (6%)	2 (11%)	1^c^
Abscess size, mm, median (IQR)	51 (44–66)	60 (52–88)	0.007^b^	58 (50–66)	58 (50–67)	0.95^b^
Charlson Comobidity Index, median (IQR)	2 (1–4)	2 (1–4)	0.94^b^	3 (1–6)	2 (1–4)	0.25^b^
Corticosteroid medication	11 (14%)	4 (20%)	0.5^c^	5 (28%)	4 (22%)	1^c^
Outcomes						
Overall failure	21 (27%)	7 (35%)	0.46^a^	8 (44%)	6 (33%)	0.49^a^
30-day mortality	4 (5%)	1 (5%)	1^c^	1 (6%)	1 (6%)	1^c^
Need of emergency surgery during initial admission or within 30 days	13 (17%)	6 (30%)	0.21^c^	5 (28%)	5 (28%)	1^a^
Readmission within 30 days of discharge	6 (14%)	2 (10%)	0.66^c^	3 (17%)	2 (11%)	1^c^
Recurrence of diverticulitis during follow-up^d^	20 (32%), *n* = 62^e^	1 (8%), *n* = 13^e^	0.1^c^	2 (17%), *n* = 12^e^	1 (8%), *n* = 12^e^	1^c^
Complicated recurrence^d^	13 (21%), *n* = 62^e^	1 (8%), *n* = 13^e^	0.44^c^	1 (8%), *n* = 12^e^	1 (8%), *n* = 12^e^	1^c^
Sigma resection later than 30 days after discharge^d^	29 (47%), *n* = 62^e^	9 (69%), *n* = 13^e^	0.14^a^	8 (44%), *n* = 12^e^	9 (50%), *n* = 12^e^	1^c^
Temporary stoma	4 (5%)	1 (5%)	1^c^	2 (17%), *n* = 12^e^	1 (8%), *n* = 12^e^	1^c^
Permanent stoma	4 (5%)	1 (5%)	1^c^	1 (8%), *n* = 12^e^	1 (8%), *n* = 12^e^	1^c^
Hospital stay, days, median (IQR)	4 (3–8)	7 (3–13)	0.17^b^	6 (3–10)	6 (3–12)	0.73^b^

*WBC* white blood cell count, *IQR* interquartile range, *CRP* C-reactive protein

^a^*χ*^2^ test

^b^Mann-Whitney *U* test

^c^Fisher’s exact test

^d^Median follow-up 71 months (IQR 46–100 months) for all patients

^e^Patients who died within 30 days or had emergency surgery during initial admission or within 30 days were removed from the total

A percutaneous drain was inserted with CT guidance for two patients, and ultrasound was used for the rest. Two (8%) patients developed an enterocutaneous fistula as a complication of percutaneous drainage. Both patients underwent sigmoidectomy, one 22 days and the other 10 days after admission. Microbiological samples collected from drained abscesses led to a change in antibiotics regimen for 4 of the 26 drained patients (15%).

To minimize the selection bias, the patients were matched in antibiotics and drainage group 1:1 by the closest abscess size. Patients without a match within 5 mm in abscess size were excluded from the analyses. In cases of two potential equal matches for the abscess size, CRP level functioned as a secondary matching criterion (without any maximum threshold for difference). Two patients in the drainage group had no match, and 18 patients were selected in each group. There were no statistically significant differences between the antibiotics and drainage groups in basic characteristics or outcomes (Table 2).

Parameters available on admission were used to identify the independent risk factors for failure of antibiotic treatment. Parameters that had a significance of *p* < 0.2 (Table 3) in univariate analysis were included in the multivariate logistic regression model using backward stepwise selection (likelihood ratio). Temperature and mean arterial pressure were excluded due to the clinically insignificant difference between the groups. Optimal cutoff points for white blood cell count (WBC) (14.8 × 10^9^/l, rounded to 15.0 × 10^9^/l), CRP (174 mg/l, rounded to 175 mg/l), and abscess size (47 mm, rounded to 50 mm) were determined by maximum value of Youden’s index for receiver operating characteristic (ROC) curve. According to multivariate analysis, WBC ≥ 15.0 × 10^9^/l, abscess diameter ≥ 50 mm, and the use of corticosteroid medication were independent risk factors for failure of antibiotic treatment (Table 3). The number of independent risk factor increased the odds ratio for failure (Table 4). The area under ROC curve for the model using these factors was 0.77 (95% confidence interval 0.68–0.87), and Nagelkerke *R*^2^ was 0.23. Univariate analysis did not identify any statistically significant risk factors for failure of drainage treatment (Table 3).

**Table 3:** Comparison of patients with antibiotics or drainage treatment regarding parameters available on admission

	Antibiotics success (*n* = 150)	Antibiotics failure (*n* = 29)	Univariate *p*	Multivariate OR (95% CI)	Drainage success (*n* = 18)	Drainage failure (*n* = 8)	*p* value
Sex female	93 (62%)	20 (69%)	0.53^a^		7 (39%)	4 (50%)	0.68^c^
Age, years, median (IQR)	61 (50–71)	60 (53–77)	0.63^b^		60 (53–71)	55 (35–69)	0.29^b^
WBC, ×10^9^/l, median (IQR)	11.6 (9.3–14.5)	15.2 (11.2–18.8)	0.001^b^		11.1 (8.6–14.8)	11. (8.3–15.6)	0.82^b^
WBC ≥ 15.0	32 (21%)	15 (52%)	0.002^a^	3.3 (1.3–8.0)	3 (17%)	2 (25%)	0.63^c^
CRP, mg/l, median (IQR)	123 (82–169)	181 (92–272)	0.009^b^		126 (115–140)	122 (31–297)	0.89^b^
CRP over ≥175	35 (23%)	15 (52%)	0.003^a^	NS	6 (33%)	4 (50%)	0.66^c^
Abscess diameter, mm, median (IQR)	33 (22–45)	50 (36–76)	< 0.001^b^		59 (49–90)	67 (54–88)	0.61^b^
Abscess diameter ≥ 50 mm	29 (19%)	16 (55%)	< 0.001^a^	3.7 (1.5–8.9)	14 (78%)	7 (89%)	1^c^
Hemoglobin, g/l, median (IQR)	131 (123–140)	126 (113–140)	0.25^b^		123 (115–140)	121 (114–143)	0.98^b^
Anemic^d^	32 (21%)	11 (38%)	0.06^a^	NS	10 (56%)	4 (50%)	1^c^
Temperature on admission, °C, median (IQR)	37.5 (37.1–37.9), *n* = 146	37.3 (36.7–37.8), *n* = 27	0.14^b^		37.3 (36.9–37.6), *n* = 17	37.1 (36.4–38.1)	0.8^b^
MAP on admission, mmHg, median (IQR)	99 (92–108), *n* = 146	95 (87–104), *n* = 27	0.07 ^b^		99 (91–111), *n* = 17	98 (87–113)	0.93^b^
Multiple abscesses	23 (15%)	3 (10%)	0.77^c^		2 (11%)	1 (13%)	1^c^
Intra-abdominal fluid in the fossa douglas	20 (13%)	4 (14%)	1^c^		0	1 (13%)	0.31^c^
Charlson Comorbidity Index, median (IQR)	2 (1–3)	3 (1–5)	0.08^b^	NS	2 (1–5)	2 (0–4)	0.5^b^
Corticosteroid medication	10 (7%)	7 (24%)	0.009^c^	3.9 (1.2–12.4)	4 (22%)	2 (25%)	1^c^
Any earlier diverticulitis	39 (26%)	10 (35%)	0.37^c^		6 (33%)	5 (63%)	0.22^c^
Earlier complicated diverticulitis	6 (4%)	3 (10%)	0.16^c^	NS	2 (11%)	4 (50%)	0.051^c^
Earlier abdominal surgery^e^	54 (36%)	14 (48%)	0.22^a^		5 (28%)	1 (13%)	0.63^c^
No guarding on admission	42 (28%)	12 (41%)	0.19^a^	NS	7 (39%)	6 (75%)	0.202^c^
Intra-abdominal or retroperitoneal air in CT (non-pericolic)	13 (9%)	6 (21%)	0.09^c^	NS	2 (11%)	1 (13%)	1^c^
Pelvic abscess	33 (22%)	12 (41%)	0.04^a^	NS	7 (39%)	4 (50%)	0.68^c^
Initially treated with antibiotics only	**–**	**–**			4 (22%)	1 (13%)	1^c^

Patients whose treatment was started with antibiotics alone are also included in the drainage group. Parameters with univariate *p* < 0.2 were selected for the multivariate logistic regression model. Temperature and MAP were left out of the model due to clinically non-significant differences

*OR* odds ratio, *CI* confidence interval, *IQR* interquartile range, *WBC* white blood cell count, *CRP* C-reactive protein, *NS* not selected in backward stepwise selection (likelihood ratio), *CT* computer tomography, *MAP* mean arterial pressure

^a^*χ*^2^ test

^b^Mann-Whitney *U* test

^c^Fisher’s exact test

^d^Hemoglobin < 117 g/l for females or < 134 g/l for males

^e^Including appendectomy, cholecystectomy, hysterectomy, gastrectomy, and resections of bowel

**Table 4:** Odds ratios for the failure of treatment with antibiotics alone compared to zero risk factors

Number of risk factors	Odds ratio	95% confidence interval	*p* value	Number of patients treated with antibiotics alone, *n*	Number of patients with failure of antibiotic treatment, *n* (%)
0	Reference			101	5 (5%)
1	5.6	1.8–17.1	0.003	49	11 (22%)
≥ 2	15.6	4.9–49.7	< 0.001	29	13 (45%)

Risk factors for failure of treatment were corticosteroid use, abscess diameter ≥ 50 mm, and white blood cell count ≥ 15.0 × 10^9^/l.

## Discussion

Abscess size has a drastic effect on the choice and success of treatment of diverticular abscesses. Abscesses under 40 mm were mostly treated with only antibiotics with a high success rate (87%). This reflects current international guidelines for the treatment of small diverticular abscesses [11]. Patients with an abscess larger than 80 mm often had conditions requiring immediate surgery, such as peritonitis or free air in CT scan, and surgery was the most common first-line treatment (43%) in this group. Half of those, who were initially treated conservatively, required surgery within 30 days. Percutaneous drainage was attempted for 35% of patients with abscess ≥ 40 mm, who did not undergo surgery as the first-line treatment. However, due to technical difficulties, only 18% were successfully drained. Treatment with antibiotics alone decreased as abscess size increased.

Percutaneous drainage combined with antibiotics as a treatment for abscess did not seem to be superior when compared to treatment by only antibiotics. Our data showed no differences in the failure rate, 30-day mortality, need of emergency surgery, permanent stoma, recurrence, or length of stay even between the groups of matched patients. WBC count ≥ 15.0 × 10^9^/l, abscess diameter ≥ 50 mm, and corticosteroid medication were independent risk factors for failure of treatment with antibiotics alone.

Over half of the patients in our study required surgery for abscesses ≥ 60 mm, and in 42%, surgery was the first-line treatment. The success rate of surgery for these patients was 69% (no reoperation or death within 30 days). Emergency surgery is not recommended as the first-line treatment for abscesses due to high mortality [1, 3, 11, 14]. However, the excess mortality might be due to the factors unrelated to surgery such as comorbidities or sepsis. Selected patients might benefit from early operative intervention. Previous studies have usually excluded patients treated operatively as the first-line treatment. Only Devaraj et al. [4] and Garfinkle et al. [15] include them. These studies report an overall emergency surgery rate of 12% and 23%, respectively. Neither reports the number separately for large abscesses. In studies by Ambrosetti et al. [5] and Kaiser et al. [16], emergency surgery was required for 15% vs. 39% and 19% vs. 32% in Hinchey Ib and Hinchey II diverticulitis, respectively. These studies do not directly report abscess size for operated patients, but pelvic abscesses are generally larger than pericolic. In our data, over 60% of abscesses ≥ 60 mm were pelvic while only 10% of abscesses under 40 mm were pelvic.

Only a few studies compare the treatment of large abscesses between percutaneous drainage and antibiotics, and all of them are retrospective series. A study of patients with Hinchey stage II diverticulitis found no differences between the drainage group (*n* = 34) and antibiotics group (*n* = 32) in overall failure (33% vs. 19%, respectively, *p* = 0.26) or emergency surgeries (29% and 16%, respectively, *p* = 0.24) [7]. However, the drainage group had significantly larger abscess diameter median (6 cm vs. 4 cm). Elagili et al. [9] compared the treatment in patients with diverticular abscess of ≥ 3 cm. In the study, 32 patients were initially treated with antibiotics alone and 114 with percutaneous drainage. The study found no significant differences between the drainage and antibiotics groups in need of urgent surgery (18% vs. 25%, respectively, *p* = 0.21). The authors suggested that antibiotics without percutaneous drainage could be used as the initial treatment for selected patients even with large diverticular abscesses. The abscess size was larger in the drainage group (71 mm vs. 59 mm). Garfinkle et al. [15] evaluated the long-term safety of non-operative treatment for diverticular abscess. The 73 patients in this retrospective study, of which 33 underwent percutaneous drainage, had low incidences of future emergency operations (2.7% during follow-up of 62 months). However, retrospective study of 185 conservatively managed patients, of which 31% were treated by drainage, found that 28% of the patients required emergency surgery during recurrence of diverticulitis [4]. Successful drainage did not seem to lower the complication rates or recurrences. A recently published article by Lambrichts et al. [17] is the only multi-center study, which compares the treatment with antibiotics alone to percutaneous drainage in Hinchey Ib and II diverticulitis. Of overall 447 patients, 332 (74.3%) were treated with antibiotics alone. Short-term failure rates for Hinchey Ib (22.3% vs. 33%) and Hinchey II (25.9% vs. 36%) did not differ for antibiotics alone and percutaneous drainage. The choice of treatment strategy was not an independent risk factor for failure of treatment in multivariate analysis.

A systematic review about the treatment of large diverticular abscesses found treatment failure to be 19–21% regardless of treatment choice [14]. Recurrence of diverticulitis during follow-up was lower for patients treated with drainage than for those treated with antibiotics (15% vs. 25%). The pooled average for complication percentage of percutaneous drainage was 2.5% (range 0–12.5%). Majority of the complications were enterocutaneous fistulas or small bowel lesions and were treated conservatively [14].

The overall failure rates in our study, 35% for the drainage group and 27% for the antibiotics group, are comparable to previous studies. Although limited by their retrospective nature and small cohort sizes, all the studies have comparable results. Percutaneous drainage offered no clear advantages in the short- or long-term success of the treatment. However, retrospective studies are susceptible to selection bias. It is possible that physicians treated patients with a worse clinical condition more actively, and therefore, they were more likely to receive drain.

Complications of percutaneous drainage are inevitable, as they are for any invasive procedure. In our study, two (8%) drained patients developed an enterocutaneous fistula and both later underwent sigmoidectomy. Therefore, the advantages and disadvantages should be carefully considered. Drainage does not seem to decrease treatment failure. However, drainage or aspiration of an abscess enables antibiotic susceptibility test, which could result in a change of antibiotics as it did in 15% of patients in our series.

There are several limitations to this study. This is a retrospective study with all the limitations inherent in the design. In most cases, the exact reason for placing drain cannot be assessed. Also, the sample size is relatively small. Most other studies comparing antibiotics treatment with percutaneous drainage have these same limitations. Data about recurrences was only collected from our institution’s patient records. Mild recurrences which were diagnosed and treated in primary care without CT imaging or need of hospitalization were not included in recurrences. Death or moving away from the referral area terminated follow-up.

The rarity of large diverticular abscesses amenable for drainage presents difficulties for conducting prospective studies. Our data from all CT-imaged diverticulitis patients presented in our hospital’s emergency department over 8 years contains only 21 patients, who received percutaneous drainage as their first-line treatment. Seven patients with only percutaneous aspiration were included in the drainage group, and one might argue that these patients did not receive proper drainage. However, it is unclear whether aspiration is as effective as drainage [3]. As there is no evidence for drain irrigation regimes or discontinuation of the drainage [3], the drains are usually removed at our institution once the abscess is emptied and the drains do not produce pus anymore.

## Conclusions

In conclusion, percutaneous drainage combined with antibiotics is not superior to antibiotics only in terms of treatment failure, recurrence of diverticulitis, or incidence of elective sigmoid resections regardless of the abscess size. Unless emergency surgery is needed, antibiotics could be considered as the primary treatment. Percutaneous drainage is an invasive procedure and does not seem to improve treatment results. Therefore, drainage should be considered when it is technically feasible and antibiotic treatment does not improve the patient. A prospective randomized study is needed to comprehensibly evaluate the advantages and disadvantages of percutaneous drainage in diverticular abscesses. This will be challenging to commence because of the rarity of the disease and would probably need an international collaboration to be successfully carried out.

## Data Availability

The datasets cannot be made publicly available, and restrictions apply to the availability of these data. Data can be requested from the authors and will require permission from the Helsinki University Hospital.

## Abbreviations

CRP: C-reactive protein
CT: Computed tomography
IQR: Interquartile range
ROC: Receiver operating characteristic
WBC: White blood cell count
